# Effects of Omega-3 Fatty Acid Supplementation on Diabetic Nephropathy Progression in Patients with Diabetes and Hypertriglyceridemia

**DOI:** 10.1371/journal.pone.0154683

**Published:** 2016-05-02

**Authors:** Eugene Han, Yujung Yun, Gyuri Kim, Yong-ho Lee, Hye Jin Wang, Byung-Wan Lee, Bong Soo Cha, Beom Seok Kim, Eun Seok Kang

**Affiliations:** 1 Division of Endocrinology and Metabolism, Department of Internal Medicine, Yonsei University College of Medicine, Seoul, Korea; 2 Brain Korea 21 Project for Medical Science, Yonsei University College of Medicine, Seoul, Korea; 3 Institute of Endocrine Research, Yonsei University College of Medicine, Seoul, Korea; 4 Division of Nephrology, Department of Internal Medicine, Yonsei University College of Medicine, Seoul, Korea; 5 Graduate school, Yonsei University College of Medicine, Seoul, Korea; Max Delbrueck Center for Molecular Medicine, GERMANY

## Abstract

Beneficial effects of omega-3 fatty acid (O3FA) supplementation in a wide range of disease condition have been well studied. However, there is limited information regarding the effects of O3FAs on chronic kidney disease (CKD), especially in diabetic nephropathy (DN) with hypertriglyceridemia. We investigate whether O3FA supplementation could help maintain renal function in patients with diabetes and hypertriglyceridemia. Total 344 type 2 diabetic patients with a history of O3FA supplementation for managing hypertriglyceridemia were included. Reduction in urine albumin to creatinine ratio (ACR) and glomerular filtrate rate (GFR) were examined. Subgroup analyses were stratified according to the daily O3FA doses. Serum total cholesterol, triglyceride, and urine ACR significantly reduced after O3FA supplementation. Overall, 172 (50.0%) patients did not experience renal function loss, and 125 (36.3%) patients had a GFR with a positive slope. The patients treated with O3FAs at 4g/day showed greater maintenance in renal function than those treated with lower dosages (p < 0.001). This dose dependent effect remains significant after adjustment for multiple variables. O3FA supplementation in diabetic patients with hypertriglyceridemia shows benefits of reducing albuminuria and maintaining renal function. The effects are dependent on the dose of daily O3FA supplementation.

## Introduction

Chronic kidney disease (CKD) is defined as kidney dysfunction present for > 3 months, with a glomerular filtration rate (GFR) < 60 mL/min/1.73 m^2^ [1]. Many studies have reported that both lower GFR and greater albuminuria are independently related to the rates of end-stage renal disease (ESRD), cardiovascular events, and mortality. Albuminuria severity is strongly predictive of poor outcomes at all levels of GFR. More than 40% of subjects with type 2 diabetes mellitus (DM) develop diabetic nephropathy (DN) [2], which has become the leading cause of ESRD [3]. DN is characterized by abnormal urinary albumin excretion progressing from microalbuminuria (30–300 μg/min) to macroalbuminuria (> 300 μg/min) [1]. In addition, GFR is known to decrease with age, at less than 1 ml/min/1.73 m^2^/year, in healthy individuals [4]; this is accelerated in DN to 5.2 ml/min/1.73 m^2^/year [5]. Nevertheless, early screening and limited intervention can prevent or delay DN progression in diabetic patients with microalbuminuria [6].

Omega-3 fatty acids (O3FAs) are polyunsaturated fatty acids (PUFAs) derived from fish oil; the three types are α-linoleic acid (ALA), eicosapentaenoic acid (EPA) and docosahexaenoic acid (DHA). These are essential fatty acids, and small amounts of dietary O3FAs enable normal growth. O3FAs also reduce blood triglyceride levels [7–9]. Although somewhat controversial, regular O3FA supplementation may reduce the risks of myocardial infarction and sudden cardiac death [10]. Some evidence suggests that it may improve blood circulation; increase the breakdown of fibrin, a compound involved in clot and scar formation; and reduce blood pressure and heart rate [11–13]. Hypertension is a major risk factor for CKD development, and it is expected that O3FAs could inhibit CKD progression due to their anti-hypertensive effect.

Numerous studies have evaluated the potential beneficial effects of O3FAs on inflammatory, autoimmune, and renal diseases [14, 15]. O3FAs can reduce proteinuria in patients with chronic glomerular disease and slow immunoglobulin A (IgA) nephropathy [16]. Due to their anti-inflammatory effects, O3FAs have been suggested to protect against kidney damage. An animal model study showed that O3FA supplementation reduced renal inflammation and fibrosis [17]. In addition, a large community-based cohort study reported that increased dietary intake of long-chain n-3 PUFA and fish reduces CKD prevalence [18].

However, there is limited information regarding the effects of O3FAs on CKD, especially in patients with DN. The role of O3FAs in DN has not been thoroughly investigated. Therefore, we performed a retrospective cohort study to examine whether O3FA supplementation could preserve renal function in diabetic subjects.

## Materials and Methods

In this retrospective study, subjects were identified by reviewing patient case notes using electronic medical records at Severance Hospital, a tertiary university hospital in Korea. We included 344 patients ≥ 20 years old with a history of O3FA (highly purified ethyl ester concentrate of EPA and DHA) supplementation for managing hypertriglyceridemia. Diabetes mellitus (DM) was defined according to the International Classification of Diseases 10th revision (ICD 10). Only new users who had not used O3FAs in a preceding year were enrolled. The mean supplement duration (‘after treatment’ period) was 1.9 ± 1.6 years (median 1.4 years). O3FA was used for at least 3 months. Subjects were excluded if they fulfilled any one of the following criteria: (1) kidney transplanted patient; (2) dialysis patient; (3) intrinsic renal disease (nephritis or nephrotic syndrome) patient; (4) acute renal failure due to septic shock, contrast agents, or drugs; and (5) postrenal disease patient. The patient records was anonymized and de-identified prior to analysis. The study protocol received ethical approval by the institutional review board at the Yonsei University College of Medicine (4-2014-0273).

### Clinical and laboratory parameters

We evaluated the effects of O3FAs on DN and albuminuria. The dose-effect relationships between O3FAs and albuminuria, renal function were also analyzed. Baseline estimated glomerular filtration rates (GFRs), and spot urine ACRs were measured at the start of O3FA administration. Overnight 8-hours fasting random spot urine samples were collected, and urine ACR was measured with an immunoturbidimetric method. After O3FA treatment (median 1.4 years), we evaluated changes in these laboratory parameters and also examined clinical parameters, blood glucose, lipid profiles. GFR was calculated using the Chronic Kidney Disease Epidemiology Collaboration (CKD-EPI) equation [19]. Body mass index (BMI) was defined as body weight (kg) divided by the square of height (m^2^).

### Data analysis

The primary outcome was renal function changes during O3FAs supplementation, specifically in GFR and urine ACR. We compared the annual rates of GFR decline among three dose categories: 1 g/day, 2 g/day, and 4 g/day. Subgroup analyses were stratified according to daily O3FA doses; daily doses of 4 g O3FA were grouped into the high-dose group, and those <4 g were placed in the low-dose group. In addition, the proportion of subjects with preserved GFR was analyzed according to O3FA dosages.

### Statistical analysis

The data are reported as mean ± standard deviation (SD), and the real numbers of participants with the percentages follow in parentheses. Simple comparisons of continuous variables within or between sub-groups were made with Student’s *t*-tests. Paired data measured at baseline and after O3FA supplementation were compared using paired *t* tests. Since urine ACR, total cholesterol, triglyceride, high density lipoprotein (HDL) cholesterol, and high sensitivity C-reactive protein (hsCRP) values were not normally distributed, analyses were performed using log- and back-transformed data. Chi-square tests were used to examine the relationships between multiple variables. The relative factor for GFR decline was obtained using univariate and multiple logistic regressions, and the risk is reported in the form of odds ratios (ORs) and 95% confidence intervals (CIs). All statistical analyses were performed using the SPSS statistical analysis program (SPSS vs. 20.0, IBM Corp, Armonk, NY). For all tests, p < 0.05 was considered statistically significant.

## Results

### Subject clinical characteristics

Table 1 shows the baseline clinical characteristics of patients. A total of 344 patients (244 males, 100 females) were recruited with a mean O3FA supplement duration of 1.9 ± 1.6 years. More than 90% of patients were treated with O3FA < 4 g/day. Three-quarters of patients had hypertension that was relatively well-controlled (mean systolic blood pressure 127.8 ± 15.1 mmHg, mean diastolic blood pressure 74.9 ± 9.6 mmHg). About 72% of these patients were using angiotensin converting enzyme (ACE) inhibitors or angiotensin receptor blockers (ARBs).

**Table 1:** Baseline characteristics of study subjects.

Baseline Parameters	Values
Male gender, N (%)	244 (70.9)
Age (year)	56.8 ± 11.8
DM duration (year)	6.5 ± 7.6
O3FAs dosage	1875.0 ± 796.5
1 g/day, N (%)	103 (29.9)
2 g/day, N (%)	211 (61.3)
4 g/day, N (%)	30 (8.7)
BMI (kg/m^2^)	26.0 ± 3.4
Smoking, N (%)	152 (44.2)
Drinking alcohol, N (%)	147 (42.7)
Hypertension, N (%)	256 (74.4)
Systolic blood pressure (mmHg)	127.8 ± 15.1
Diastolic blood pressure (mmHg)	74.9 ± 9.6
Medications, N (%)	
ACEi/ARB	247 (71.8)
CCB	151 (43.9)
SU	154 (44.8)
Metformin	268 (77.9)
DPP4i	126 (36.6)
TZD	46 (13.4)
Insulin	59 (17.2)
Fenofibrate	54 (15.7)
Statin	211 (61.3)
Ezetimibe	42 (12.2)

Data are presented as N (%) or mean ± SD. DM, diabetes mellitus; O3FAs, omega-3 fatty acids; BMI, body mass index; ACEi, angiotensin-converting enzyme inhibitor; ARB, angiotensin II receptor blocker; CCB, calcium channel blocker; SU, sulfonylurea; DPP4i, dipeptidyl peptidase-4 inhibitor; TZD, thiazolidinedione.

### Effects of O3FA supplementation on renal function loss

Urine ACR was significantly decreased after O3FA supplementation (from 475.8 ± 1235.9 mg/g to 385.6 ± 1067.9 mg/g, Δ = -72.1 ± 507.6 mg/g, p = 0.003; Table 2). However, a significant GFR decline was observed during the study periods (Table 2). The mean annual GFR loss was 2.6 ± 12.7 mL/min/1.73 m^2^ (median value 0.9 mL/min/1.73 m^2^). GFR did not decrease in 172 (50.0%) patients and increased in 125 (36.3%) patients after O3FA supplementation. We also analyzed the baseline characteristics of study groups stratified by whether GFR was preserved (S1 Table). There were no significant differences in baseline parameters according to the presence of GFR loss in renal function, except for diabetes duration, O3FAs dose, systolic blood pressure, and urine ACR. The shorter diabetes duration and lower urine ACR the patients had, the greater the benefit to preserving GFR was observed (S1 Table). Greater maintenance of renal function was observed in the high dose group (Table 3). The annual decline in GFR ranged from 3.6 ± 10.8 mL/min/1.73 m^2^ in the 1 g/day group to 2.6 ± 13.7 mL/min/1.73 m^2^ in the 2 g/day group. For the 4 g/day group, the GFR decline was -0.3 ± 11.5 mL/min/1.73 m^2^. However, there was no statistically significant difference in annual GFR loss among these three groups. The proportions of patients without GFR decline were 37.9%, 54.0%, and 63.3% in the 1 g/day, 2 g/day and 4 g/day groups, respectively (p = 0.006 for trend). Univariate logistic regression analysis was performed to identify variables associated with kidney function loss (Table 3). In terms of GFR loss, O3FA doses of 2 g/day (OR = 0.52, 95% CI 0.32–0.84, p = 0.008) and 4 g/day (OR = 0.35, 95% CI 0.15–0.82, p = 0.015) were beneficial, whereas longer diabetes duration, higher systolic blood pressure, and fenofibrate use had a negative effect (Table 3). Next, we sought to confirm the efficacy of high-dose O3FAs on DN. As shown in S2 Table, patients treated with high doses of O3FAs tended to have increased baseline triglycerides and fenofibrate use. Multivariable logistic regression models were applied to adjust for other conventional confounding covariates that affect DN, along with fenofibrate, statin use, and triglycerides levels (Table 4). O3FA at 2 g/day had a positive effect on GFR preservation (OR = 0.43, 95% CI 0.26–0.74, p <0.05), on which O3FA at 4 g/day showed a markedly higher benefit (OR = 0.20, 95% CI 0.08–0.52, p <0.05). The same trends were observed in patients without fenofibrate use (S3 Table).

**Table 2:** Changes in clinical parameters before and after O3FAs supplement.

Parameters	Before	After	Difference	P
Urine ACR (mg/g)	475.8 ± 1235.9	385.6 ± 1067.9	-72.1 ± 507.6	<0.001
Urine ACR, log- transformed	4.0 ± 2.0	3.8 ± 2.0		0.003
GFR (mL/min/1.73 m^2^)	76.1 ± 25.9	74.4 ± 27.9	-1.8 ± 11.2	0.004
Fasting blood glucose (mg/dL)	141.2 ± 50.9	129.7 ± 36.6	-11.5 ± 3.0	<0.001
Postprandial blood glucose (mg/dL)	223.6 ± 84.7	208.2 ± 66.1	-15.3 ± 91.4	0.020
HbA1c (%)	7.8 ± 6.3	7.1 ± 1.1	-0.6 ± 6.3	0.084
Total cholesterol (mg/dL)	187.6 ± 55.9	158.8 ± 41.1	-28.8 ± 55.3	<0.001
Total cholesterol, log-transformed	5.2 ± 0.3	5.0 ± 0.3		<0.001
Triglyceride (mg/dL)	417.9 ± 646.5	227.4 ± 172.3	-190.4 ± 633.1	<0.001
Triglyceride, log-transformed	5.8 ± 0.7	5.2 ± 0.6		<0.001
HDL cholesterol (mg/dL)	41.1 ± 22.1	40.4 ± 12.7	-0.7 ± 23.9	0.573
HDL cholesterol, log-transformed	3.7 ± 0.3	3.7 ± 0.3		0.975
Uric acid (mg/dL)	6.02 ± 1.74	5.80 ± 1.59	-0.21 ± 1.42	0.006
hsCRP (mg/L)	5.9 ± 14.4	2.4 ± 8.3	-3.5 ± 15.3	0.004
hsCRP, log transformed	0.5 ± 1.4	0.1 ± 1.0		<0.001

Data are presented as N (%) or mean ± SD. O3FA, omega-3 fatty acid; ACR, albumin to creatinine ratio; GFR, glomerular filtration rate; HbA1c, glycated hemoglobin; HDL cholesterol, high-density lipoprotein cholesterol; hsCRP, high sensitivity C-reactive protein.

**Table 3:** Univariate logistic regression analysis to determine variables associated with GFR decline.

	OR	95% CI	P
O3FAs dosage			
1 g/day	Reference		
2 g/day	0.52	0.32–0.84	0.008
4 g/day	0.35	0.15–0.82	0.015
Age (year)	0.99	0.98–1.01	0.505
Sex (female)	1.12	0.70–1.79	0.635
Diabetes duration (year)	1.05	1.02–1.09	0.001
Systolic blood pressure (mmHg)	1.01	1.00–1.03	0.037
ACEi/ARB use	1.14	0.72–1.79	0.564
Statin use	1.08	0.70–1.67	0.740
Fenofibrate use	2.04	1.12–3.70	0.019
Fasting blood glucose	1.00	1.00–1.01	0.056
HbA1c	0.99	0.96–1.02	0.452
Baseline GFR	0.66	0.41–1.08	0.097
Baseline triglyceride	1.15	0.84–1.59	0.371

O3FA, omega-3 fatty acid; OR, odds ratio; 95% CI, 95% confidence interval; ACEi, angiotensin-converting enzyme inhibitor; ARB, angiotensin II receptor blocker; HbA1c, glycated hemoglobin; GFR, glomerular filtration rate.

**Table 4:** Multiple logistic regression analysis to determine variables associated with GFR decline.

	O3FA 2 g/day		O3FA 4 g/day	
	OR (95% CI)	P	OR (95% CI)	P
Model 1	0.51 (0.32–0.83)	0.007	0.34 (0.15–0.79)	0.013
Model 2	0.47 (0.28–0.77)	0.003	0.21 (0.08–0.54)	0.001
Model 3	0.43 (0.26–0.74)	0.002	0.20 (0.08–0.52)	0.001

Reference group = patients with O3FA 1g/day; Model 1: adjusted for age and sex; Model 2: adjusted for age, sex, diabetes duration, body mass index, systolic blood pressure, angiotensin-converting enzyme inhibitor/angiotensin II receptor, and statin and fenofibrate use; Model 3: adjusted for age, sex, diabetes duration, body mass index, systolic blood pressure, angiotensin-converting enzyme inhibitor/angiotensin II receptor, statin, fenofibrate, fasting blood glucose, baseline GFR, triglycerides, and total cholesterol. GFR, glomerular filtration rate; O3FAs, omega-3 fatty acids; OR, odds ratio; 95% CI, 95% confidence interval.

### Effects of O3FA supplementations on metabolic parameters

At enrollment, 61.3% of patients were using statins, and 15.7% used fenofibrate. Serum triglyceride levels were 46% lower after O3FA supplementation (p < 0.001), and total cholesterol decreased by 19% (p < 0.001). There were no significant changes in HDL cholesterol after O3FA treatment (Table 2). However, uric acid levels and hsCRP were significantly reduced (All ps < 0.05, Table 2).

## Discussion

The major finding of this study is that O3FA supplementation may help with preserving renal function in type 2 diabetes patients with hypertriglyceridemia. O3FA influenced both urine ACR and GFR, indicating a protective effect for O3FA on DN. In addition, the effects were dependent on the dose of daily O3FA supplementation. Although GFR decline was observed in our study population, the mean annual rate of GFR decline was relatively slower than that reported in a previous study [5].

A possible association between O3FA supplementation and albuminuria reduction was first noticed after EPA was found to improve albuminuria in diabetic patients [20]. After EPA was administered to 16 diabetic patients (5 type 1 and 11 type 2) for 6 months, albuminuria measured with spot urine was significantly improved without altering blood glucose levels, body weight, or blood pressure. A large nested control study including 1,150 type 1 DM subjects showed that higher n-3 PUFA intake was related to a lower risk of microalbuminuria [21]. There are also some studies supporting the hypothesis that O3FA supplementation slows the progression of IgA nephropathy. For IgA nephropathy patients with persistent proteinuria, fish oil supplementation for 2 years delayed nephropathy progression compared to olive oil supplementation, and this effect appeared to be independent of reduced blood pressure [16]. A meta-analysis showed n-3 PUFA reduces urine protein excretion among IgA nephropathy, DM, and lupus nephritis patient populations [22]. In addition, O3FA supplementation reportedly reduces DM complications [23]. The pathway of beneficial effects of O3FA in kidney has been studied in animal experiments: improvement of dyslipidemia and attenuation of inflammation. Streptozotocin-induced diabetic rats fed a high n-3 PUFA diet were more resistant to renal disease development [24]. N-3 PUFA treatment attenuated diabetes-associated glomerulosclerosis and tubulointerstitial fibrosis [24]. In addition, after n-3 PUFA supplementation, expression levels of transforming growth factor (TGF)-β, which is a marker of end-organ complications associated with DM, and those of interleukin (IL)-6 and monocyte chemoattractant protein (MCP)-1, markers of tissue inflammation, were attenuated [24]. Similarly, in DN of type 2 diabetic KKA^y^/Ta mice model, improved urine ACR and down regulated MCP-1 levels were observed [25]. Mesangial matrix accumulation and tubule-interstitial fibrosis were attenuated in EPA-injected mice, but where were no alterations in systolic blood pressure or fasting blood glucose levels [25]. Moreover, O3FA fed db/db mice showed reductions in renal triglycerides and renal SREBP-1 expression, as well as attenuation of podocyte injury [26]. In our study, although levels were only measured in half of the patients hsCRP levels were significantly reduced after O3FAs supplementation.

The dose-related effect of O3FAs on DN has not been studied, but it has been investigated in the context of heart disease. According to the American Heart Association guideline, consuming 2 servings (8 ounces) of food containing very long-chain O3FAs reduces the risks of sudden death and death from coronary artery disease [27]. The mechanisms by which O3FAs influence cardiovascular disease include amelioration of arrhythmias, thrombosis, inflammatory responses, triglycerides, remnant lipoprotein levels, low-density lipoprotein cholesterol, atherosclerotic plaque instability, endothelial dysfunction, and blood pressure [28, 29]. Among these factors, lowered triglyceride efficacy is thought to be a dose-response effect of O3FAs [30]. In previous studies, 4 g/day of O3FAs reduced serum triglyceride levels by up to 30% [31–34]. A comparison study of O3FAs dose-response effects on triglycerides in healthy individuals demonstrated that high-dose (3.4 g/day) O3FAs significantly decreased triglyceride, whereas there were no changes in blood triglyceride levels in the placebo or low-dose (0.85 g/day) groups [35].

Studies suggest that statin could reduce albuminuria and slow down kidney function loss [36–38]. In subgroup analysis of the Greek atorvastatin and coronary heart disease evaluation study (GRACE), creatinine clearance increased in patients with statin use (4.9%, p = 0.003) [36]. Interestingly, among statin users, patients treated with atorvastatin experienced better renal outcomes, with a 12% increase in creatinine clearance (p <0.001) [36]. The renoprotective effect of statin is based on the fact that dyslipidemia and hypertension accelerate renal function loss [38]. In our study, 78 subjects were prescribed atorvastatin (37.0%) among patients treated with any statin. The proportions of those with preserved GFR were 51.1% for non-statin users and 49.3% for stain users (p = 0.740). In addition, 48.7% of atorvastatin (median dose 10 mg/day) users showed preserved GFR, which was similar to that for other statin users (49.6%) (p = 0.899). The median dose of atorvastatin in our study was 10 mg/day, which was lower than that in a previous study (24 mg/day) [36]. As the lipid lowering effect of atorvastatin depends on the daily dose [37], the difference in atorvastatin dose would lead to discordant results. Meanwhile, the association between fenofibrate, another lipid lowering drug, and renoprotective effect is controversial [39–42]. It might reduce albuminuria by antioxidant and anti-inflammatory mechanisms, in addition to a hypertriglyceridemia lowering effect [39, 40]. In an international study on type 2 diabetes patients, a fenofibrate treatment group experienced 2.6% regression or no progression in renal function deterioration, compared to a placebo group (p = 0.002) [41]. Moreover, 5-year use of fenofibrate was found to reduce albuminuria and inhibit GFR decline independently from ACE inhibitor/ARB use [39]. However, others have reported decreases in creatinine clearance and GFR with fenofibrate use [42]. In our study, as shown in univariate analysis (Table 3), fenofibrate use showed a negative effect on preserving renal function. We then conducted subgroup analysis excluding subjects with fenofibrate use. Therein, as shown in S3 Table, O3FA supplement was still associated with maintaining kidney function in a dose dependent manner.

Conversely, there are some reports that O3FA supplementation is not associated with reduced albuminuria or that this effect is not dose-dependent. A cohort study assessed both type 1 and 2 DM patients with microalbuminuria and reported that treatment with 4 g/day of O3FAs did not improve albuminuria [43]. However, the study enrolled a small number of patients, and the total treatment duration was only 12 weeks. A randomized trial assessing severe IgA nephropathy patients showed no additional benefit of high-dose O3FA supplementation [44]. In our data, O3FAs reduced urine ACR or preserved GFR was mostly observed in patients with early DN. This might explain why O3FAs did not have dose-dependent effects in patients with severe IgA nephropathy. In a randomized trial with type 2 diabetes patients, 4 g/day of n-3 PUFA supplement during 6 weeks did not show significant changes in urine ACR [45]. However, there was significant improvement in serum renal damage markers, suggesting the beneficial effect of n-3 PUFA on renal function.

Our study had several limitations. Because it was a retrospective investigation, there were many critical confounders influencing albuminuria or nephropathy. First, ACE inhibitors and ARBs are known to ameliorate albuminuria in DN. More than half of patients (71.8%) were prescribed those medications. Although there were no differences in medication use between the GFR decline progression and no-progression groups, we analyzed ACE inhibitor or ARB use as a covariate factor. Second, medication compliance was not fully considered. As this was not a prospective study, we could not assess adherence with the prescribed O3FA. The study data were based on the proposition that all the prescribed medication was administrated to the subjects. Third, hyperglycemia was improved in the course of the study. Fasting and postprandial blood glucose levels were significantly decreased after O3FAs supplementation. There is a possibility that improved hyperglycemia may contribute to renal function maintain. However, the correlation analysis showed that improvement in serum triglyceride level is more closely associated with better GFR maintain after adjustment fasting blood glucose reduction (r = 0.18, p = 0.001). Fourth, most patients enrolled in this study were relatively early stage DN, as 77.9% of patients were treated with metformin (contraindicated for advanced kidney disease patients) and > 70% of patients were normo-, or microalbuminuria or CKD stage I or, II. In addition, we did not include a control group. However, it would be immoral practice to prescribe a placebo to patients with hypertriglyceridemia, especially to subjects with serum triglyceride more than 500 mg/dl, due to the risk of acute pancreatitis.

Our study also had a major strength. We showed dose dependency in O3FAs effects on attenuating GFR loss. This is the first study reporting an association between O3FA supplementation dosages and DN. A novel finding is the identification of an effective O3FA dose in DN patients with hypertriglyceridemia. In addition, we enrolled compliant patients without other kidney diseases who were selected based on detailed reviews of their medical records.

In conclusion, our findings demonstrate that O3FA supplementation in diabetic patients with hypertriglyceridemia ameliorated urine ACR and preserved GFR. This suggests that O3FA supplementation in diabetic patients with hypertriglyceridemia could ameliorate DN progression in a dose-dependent manner. However, further randomized trials are needed to quantify this beneficial effect compared to placebo and control groups. If there is clear dose-dependent effect of O3FAs on DN, high-dose O3FA administration will likely provide a therapeutic option for high-risk diabetic patients.

## Supporting Information

S1 Dataset The anthropometric and biochemistry characteristics of the subjects. (XLSX)

S1 Table Baseline characteristics of patients preserved GFR. Data are presented as N(%) or mean ± SD. DM, diabetes mellitus; O3FAs, omega-3 fatty acid; BMI, body mass index; ACEi, angiotensin-converting enzyme inhibitor; ARB, angiotensin II receptor blocker; CCB, calcium channel blocker; SU, sulfonylurea; DPP4i, dipeptidyl peptidase-4 inhibitor; TZD, thiazolidinedione; Urine ACR, urine albumin to creatinine ratio; GFR, glomerular filtration rate; HbA1c, glycated hemoglobin; HDL cholesterol, high-density lipoprotein cholesterol; hsCRP, high sensitivity c-reactive protein.*Log transformed.
(DOCX)

S2 Table Baseline characteristics of patients according to O3FAs daily dose. Data are presented as N(%) or mean ± SD. BMI, body mass index; HDL cholesterol, high density lipoprotein cholesterol; GFR, glomerular filtration rate; ACR, albumin to creatinine ratio; ACE inhibitor, angiotensin-converting enzyme inhibitor; ARB, angiotensin II receptor blocker.*Log transformed.
(DOCX)

S3 Table Multiple logistic regression analysis to determine variables associated GFR decline in subjects without fenofibrate use. Reference group = patients with O3FA 1g/day, Model 1: adjusted for age and sex, Model 2: adjusted for age, sex, diabetes duration, body mass index, systolic blood pressure, angiotensin-converting enzyme inhibitor/angiotensin II receptor, and statin use, Model 3: adjusted for age, sex, diabetes duration, body mass index, systolic blood pressure, angiotensin-converting enzyme inhibitor/angiotensin II receptor, statin, fenofibrate, fasting blood glucose, baseline GFR, triglycerides, and total cholesterol. GFR, glomerular filtration rate; O3FAs, omega-3 fatty acids; OR, odds ratio; 95% CI, 95% confidence interval.
(DOCX)

## References

[1] Inker LA, Astor BC, Fox CH, Isakova T, Lash JP, Peralta CA, et al KDOQI US commentary on the 2012 KDIGO clinical practice guideline for the evaluation and management of CKD. Am J Kidney Dis. 2014;63(5):713–35. 10.1053/j.ajkd.2014.01.416 . 24647050

[2] Reddy MA, Tak Park J, Natarajan R. Epigenetic modifications in the pathogenesis of diabetic nephropathy. Semin Nephrol. 2013;33(4):341–53. 10.1016/j.semnephrol.2013.05.006 24011576 PMC3767931

[3] Kim SY, Jin DC, Bang BK. Current status of dialytic therapy in Korea. Nephrology (Carlton). 2003;8 Suppl:S2–9. . 15012684 10.1046/j.1440-1797.8.s.5.x

[4] Shlipak MG, Katz R, Kestenbaum B, Fried LF, Newman AB, Siscovick DS, et al Rate of kidney function decline in older adults: a comparison using creatinine and cystatin C. American journal of nephrology. 2009;30(3):171–8. 10.1159/000212381 19349699 PMC2820322

[5] Rossing K, Christensen PK, Hovind P, Tarnow L, Rossing P, Parving HH. Progression of nephropathy in type 2 diabetic patients. Kidney international. 2004;66(4):1596–605. 10.1111/j.1523-1755.2004.00925.x . 15458456

[6] Gross JL, de Azevedo MJ, Silveiro SP, Canani LH, Caramori ML, Zelmanovitz T. Diabetic nephropathy: diagnosis, prevention, and treatment. Diabetes care. 2005;28(1):164–76. . 15616252 10.2337/diacare.28.1.164

[7] Harris WS. n-3 fatty acids and serum lipoproteins: human studies. The American journal of clinical nutrition. 1997;65(5 Suppl):1645S–54S. . 9129504 10.1093/ajcn/65.5.1645S

[8] Sanders TA, Oakley FR, Miller GJ, Mitropoulos KA, Crook D, Oliver MF. Influence of n-6 versus n-3 polyunsaturated fatty acids in diets low in saturated fatty acids on plasma lipoproteins and hemostatic factors. Arterioscler Thromb Vasc Biol. 1997;17(12):3449–60. . 9437192 10.1161/01.atv.17.12.3449

[9] Davidson MH, Stein EA, Bays HE, Maki KC, Doyle RT, Shalwitz RA, et al Efficacy and tolerability of adding prescription omega-3 fatty acids 4 g/d to simvastatin 40 mg/d in hypertriglyceridemic patients: an 8-week, randomized, double-blind, placebo-controlled study. Clin Ther. 2007;29(7):1354–67. 10.1016/j.clinthera.2007.07.018 . 17825687

[10] Bucher HC, Hengstler P, Schindler C, Meier G. N-3 polyunsaturated fatty acids in coronary heart disease: a meta-analysis of randomized controlled trials. Am J Med. 2002;112(4):298–304. . 11893369 10.1016/s0002-9343(01)01114-7

[11] Morris MC, Sacks F, Rosner B. Does fish oil lower blood pressure? A meta-analysis of controlled trials. Circulation. 1993;88(2):523–33. . 8339414 10.1161/01.cir.88.2.523

[12] Mori TA, Bao DQ, Burke V, Puddey IB, Beilin LJ. Docosahexaenoic acid but not eicosapentaenoic acid lowers ambulatory blood pressure and heart rate in humans. Hypertension. 1999;34(2):253–60. . 10454450 10.1161/01.hyp.34.2.253

[13] Cicero AF, Ertek S, Borghi C. Omega-3 polyunsaturated fatty acids: their potential role in blood pressure prevention and management. Curr Vasc Pharmacol. 2009;7(3):330–7. . 19601857 10.2174/157016109788340659

[14] Wall R, Ross RP, Fitzgerald GF, Stanton C. Fatty acids from fish: the anti-inflammatory potential of long-chain omega-3 fatty acids. Nutr Rev. 2010;68(5):280–9. 10.1111/j.1753-4887.2010.00287.x . 20500789

[15] De Caterina R, Endres S, Kristensen SD, Schmidt EB. n-3 fatty acids and renal diseases. Am J Kidney Dis. 1994;24(3):397–415. . 8079965 10.1016/s0272-6386(12)80896-1

[16] Donadio JV Jr, Bergstralh EJ, Offord KP, Spencer DC, Holley KE. A controlled trial of fish oil in IgA nephropathy. Mayo Nephrology Collaborative Group. N Engl J Med. 1994;331(18):1194–9. 10.1056/NEJM199411033311804 . 7935657

[17] Baggio B, Musacchio E, Priante G. Polyunsaturated fatty acids and renal fibrosis: pathophysiologic link and potential clinical implications. J Nephrol. 2005;18(4):362–7. . 16245238

[18] Gopinath B, Harris DC, Flood VM, Burlutsky G, Mitchell P. Consumption of long-chain n-3 PUFA, alpha-linolenic acid and fish is associated with the prevalence of chronic kidney disease. Br J Nutr. 2011;105(9):1361–8. 10.1017/S0007114510005040 . 21255476

[19] Levey AS, Stevens LA, Schmid CH, Zhang YL, Castro AF 3rd, Feldman HI, et al A new equation to estimate glomerular filtration rate. Annals of internal medicine. 2009;150(9):604–12. 19414839 10.7326/0003-4819-150-9-200905050-00006 PMC2763564

[20] Hamazaki T, Takazakura E, Osawa K, Urakaze M, Yano S. Reduction in microalbuminuria in diabetics by eicosapentaenoic acid ethyl ester. Lipids. 1990;25(9):541–5. . 2250591 10.1007/BF02537161

[21] Mollsten AV, Dahlquist GG, Stattin EL, Rudberg S. Higher intakes of fish protein are related to a lower risk of microalbuminuria in young Swedish type 1 diabetic patients. Diabetes care. 2001;24(5):805–10. 10.2337/diacare.24.5.805 . 11347734

[22] Miller ER 3rd, Juraschek SP, Appel LJ, Madala M, Anderson CA, Bleys J, et al The effect of n-3 long-chain polyunsaturated fatty acid supplementation on urine protein excretion and kidney function: meta-analysis of clinical trials. The American journal of clinical nutrition. 2009;89(6):1937–45. 10.3945/ajcn.2008.26867 19403630 PMC3148029

[23] Stirban A, Nandrean S, Gotting C, Tamler R, Pop A, Negrean M, et al Effects of n-3 fatty acids on macro- and microvascular function in subjects with type 2 diabetes mellitus. The American journal of clinical nutrition. 2010;91(3):808–13. 10.3945/ajcn.2009.28374 . 20071644

[24] Garman JH, Mulroney S, Manigrasso M, Flynn E, Maric C. Omega-3 fatty acid rich diet prevents diabetic renal disease. Am J Physiol Renal Physiol. 2009;296(2):F306–16. 10.1152/ajprenal.90326.2008 19052104

[25] Hagiwara S, Makita Y, Gu L, Tanimoto M, Zhang M, Nakamura S, et al Eicosapentaenoic acid ameliorates diabetic nephropathy of type 2 diabetic KKAy/Ta mice: involvement of MCP-1 suppression and decreased ERK1/2 and p38 phosphorylation. Nephrology, dialysis, transplantation: official publication of the European Dialysis and Transplant Association—European Renal Association. 2006;21(3):605–15. 10.1093/ndt/gfi208 . 16282336

[26] Chin HJ, Fu YY, Ahn JM, Na KY, Kim YS, Kim S, et al Omacor, n-3 polyunsaturated fatty acid, attenuated albuminuria and renal dysfunction with decrease of SREBP-1 expression and triglyceride amount in the kidney of type II diabetic animals. Nephrology, dialysis, transplantation: official publication of the European Dialysis and Transplant Association—European Renal Association. 2010;25(5):1450–7. 10.1093/ndt/gfp695 . 20042400

[27] American Heart Association Nutrition C, Lichtenstein AH, Appel LJ, Brands M, Carnethon M, Daniels S, et al Diet and lifestyle recommendations revision 2006: a scientific statement from the American Heart Association Nutrition Committee. Circulation. 2006;114(1):82–96. 10.1161/CIRCULATIONAHA.106.176158 . 16785338

[28] Kris-Etherton PM, Harris WS, Appel LJ, Association AHANCAH. Omega-3 fatty acids and cardiovascular disease: new recommendations from the American Heart Association. Arterioscler Thromb Vasc Biol. 2003;23(2):151–2. . 12588750 10.1161/01.atv.0000057393.97337.ae

[29] Lee MW, Park JK, Hong JW, Kim KJ, Shin DY, Ahn CW, et al Beneficial Effects of Omega-3 Fatty Acids on Low Density Lipoprotein Particle Size in Patients with Type 2 Diabetes Already under Statin Therapy. Diabetes & metabolism journal. 2013;37(3):207–11. 10.4093/dmj.2013.37.3.207 23807924 PMC3689018

[30] Skulas-Ray AC, West SG, Davidson MH, Kris-Etherton PM. Omega-3 fatty acid concentrates in the treatment of moderate hypertriglyceridemia. Expert Opin Pharmacother. 2008;9(7):1237–48. 10.1517/14656566.9.7.1237 . 18422480

[31] Calabresi L, Donati D, Pazzucconi F, Sirtori CR, Franceschini G. Omacor in familial combined hyperlipidemia: effects on lipids and low density lipoprotein subclasses. Atherosclerosis. 2000;148(2):387–96. . 10657575 10.1016/s0021-9150(99)00267-1

[32] Calabresi L, Villa B, Canavesi M, Sirtori CR, James RW, Bernini F, et al An omega-3 polyunsaturated fatty acid concentrate increases plasma high-density lipoprotein 2 cholesterol and paraoxonase levels in patients with familial combined hyperlipidemia. Metabolism. 2004;53(2):153–8. . 14767865 10.1016/j.metabol.2003.09.007

[33] Mackness MI, Bhatnagar D, Durrington PN, Prais H, Haynes B, Morgan J, et al Effects of a new fish oil concentrate on plasma lipids and lipoproteins in patients with hypertriglyceridaemia. Eur J Clin Nutr. 1994;48(12):859–65. . 7889894

[34] Grundt H, Nilsen DW, Hetland O, Aarsland T, Baksaas I, Grande T, et al Improvement of serum lipids and blood pressure during intervention with n-3 fatty acids was not associated with changes in insulin levels in subjects with combined hyperlipidaemia. J Intern Med. 1995;237(3):249–59. . 7891046 10.1111/j.1365-2796.1995.tb01173.x

[35] Skulas-Ray AC, Kris-Etherton PM, Harris WS, Vanden Heuvel JP, Wagner PR, West SG. Dose-response effects of omega-3 fatty acids on triglycerides, inflammation, and endothelial function in healthy persons with moderate hypertriglyceridemia. The American journal of clinical nutrition. 2011;93(2):243–52. 10.3945/ajcn.110.003871 21159789 PMC3138218

[36] Athyros VG, Mikhailidis DP, Papageorgiou AA, Symeonidis AN, Pehlivanidis AN, Bouloukos VI, et al The effect of statins versus untreated dyslipidaemia on renal function in patients with coronary heart disease. A subgroup analysis of the Greek atorvastatin and coronary heart disease evaluation (GREACE) study. Journal of clinical pathology. 2004;57(7):728–34. 10.1136/jcp.2003.012989 15220366 PMC1770346

[37] Shepherd J, Kastelein JJ, Bittner V, Deedwania P, Breazna A, Dobson S, et al Intensive lipid lowering with atorvastatin in patients with coronary heart disease and chronic kidney disease: the TNT (Treating to New Targets) study. Journal of the American College of Cardiology. 2008;51(15):1448–54. 10.1016/j.jacc.2007.11.072 . 18402899

[38] Manttari M, Tiula E, Alikoski T, Manninen V. Effects of hypertension and dyslipidemia on the decline in renal function. Hypertension. 1995;26(4):670–5. . 7558229 10.1161/01.hyp.26.4.670

[39] Davis TM, Ting R, Best JD, Donoghoe MW, Drury PL, Sullivan DR, et al Effects of fenofibrate on renal function in patients with type 2 diabetes mellitus: the Fenofibrate Intervention and Event Lowering in Diabetes (FIELD) Study. Diabetologia. 2011;54(2):280–90. 10.1007/s00125-010-1951-1 . 21052978

[40] Han SH, Quon MJ, Koh KK. Beneficial vascular and metabolic effects of peroxisome proliferator-activated receptor-alpha activators. Hypertension. 2005;46(5):1086–92. 10.1161/01.HYP.0000187900.36455.4c . 16230515

[41] Keech A, Simes RJ, Barter P, Best J, Scott R, Taskinen MR, et al Effects of long-term fenofibrate therapy on cardiovascular events in 9795 people with type 2 diabetes mellitus (the FIELD study): randomised controlled trial. Lancet. 2005;366(9500):1849–61. 10.1016/S0140-6736(05)67667-2 . 16310551

[42] Forsblom C, Hiukka A, Leinonen ES, Sundvall J, Groop PH, Taskinen MR. Effects of long-term fenofibrate treatment on markers of renal function in type 2 diabetes: the FIELD Helsinki substudy. Diabetes care. 2010;33(2):215–20. 10.2337/dc09-0621 19846798 PMC2809252

[43] Lungershausen YK, Howe PR, Clifton PM, Hughes CR, Phillips P, Graham JJ, et al Evaluation of an omega-3 fatty acid supplement in diabetics with microalbuminuria. Ann N Y Acad Sci. 1997;827:369–81. . 9329768 10.1111/j.1749-6632.1997.tb51848.x

[44] Donadio JV Jr, Larson TS, Bergstralh EJ, Grande JP. A randomized trial of high-dose compared with low-dose omega-3 fatty acids in severe IgA nephropathy. J Am Soc Nephrol. 2001;12(4):791–9. . 11274240 10.1681/ASN.V124791

[45] Miller ER 3rd, Juraschek SP, Anderson CA, Guallar E, Henoch-Ryugo K, Charleston J, et al The effects of n-3 long-chain polyunsaturated fatty acid supplementation on biomarkers of kidney injury in adults with diabetes: results of the GO-FISH trial. Diabetes care. 2013;36(6):1462–9. 10.2337/dc12-1940 23275364 PMC3661851

