# Supplementary material for: Effects of Omega-3 Fatty Acid Supplementation on Diabetic Nephropathy Progression in Patients with Diabetes and Hypertriglyceridemia
Source: PLoS One. 2016 May 2;11(5):e0154683. doi: 10.1371/journal.pone.0154683 (PMC4852914; doi:10.1371/journal.pone.0154683)
Supplement: S1 Table — Data are presented as N(%) or mean ± SD. DM, diabetes mellitus; O3FAs, omega-3 fatty acid; BMI, body mass index; ACEi, angiotensin-converting enzyme inhibitor; ARB, angiotensin II receptor blocker; CCB, calcium channel blocker; SU, sulfonylurea; DPP4i, dipeptidyl peptidase-4 inhibitor; TZD, thiazolidinedione; Urine ACR, urine albumin to creatinine ratio; GFR, glomerular filtration rate; HbA1c, glycated hemoglobin; HDL cholesterol, high-density lipoprotein cholesterol; hsCRP, high sensitivity c-reactive protein.*Log transformed. (DOCX) [file pone.0154683.s002.docx]

| **S1 table. Baseline characteristics of patients with preserved GFR** | | | |
| --- | --- | --- | --- |
|  | GFR maintained | |  |
| Baseline Parameters | No (N=172) | Yes (N=172) | *P* |
| Male gender, N (%) | 120 (69.8) | 124 (72.1) | 0.635 |
| Age (year) | 56.4 ± 12.6 | 57.3 ± 11.1 | 0.506 |
| DM duration (year) | 7.9 ± 8.0 | 5.1 ± 7.1 | 0.001 |
| O3FA doses (mg/day) | 1755.8 ± 756.0 | 1994.2 ± 820.0 | 0.005 |
| BMI (kg/m^2^) | 26.3 ± 3.7 | 25.8 ± 3.1 | 0.198 |
| Smoking, N (%) | 75 (43.6) | 77 (44.8) | 0.828 |
| Drinking alcohol, N(%) | 86 (50.3) | 86 (49.7) | 0.914 |
| Hypertension, N (%) | 133 (77.3) | 123 (71.5) | 0.217 |
| Systolic blood pressure (mmHg) | 129.6 ± 14.7 | 126.2 ± 15.4 | 0.036 |
| Diastolic blood pressure (mmHg) | 76.4 ± 10.0 | 77.6 ± 9.3 | 0.232 |
| Medications, N (%) |  |  |  |
| ACEi/ARB | 126 (73.3) | 123 (71.5) | 0.718 |
| CCB | 86 (50.0) | 65 (37.8) | 0.023 |
| SU | 94 (54.7) | 60 (35.1) | <0.001 |
| Metformin | 127 (73.8) | 141 (82.5) | 0.053 |
| DPP4i | 64 (37.2) | 62 (36.3) | 0.855 |
| TZD | 31 (18.1) | 15 (8.8) | 0.011 |
| Insulin | 41 (23.8) | 18 (10.5) | 0.001 |
| Fenofibrate | 35 (20.3) | 19 (11.0) | 0.018 |
| Statin | 107 (62.2) | 104 (60.5) | 0.740 |
| Ezetimibe | 21 (12.2) | 12 (12.2) | 0.999 |
| Biochemistry |  |  |  |
| Urine ACR (mg/g) | 765.5 ± 1653.5 | 150.1 ± 373.5 | <0.001* |
| GFR (mL/min/1.73 m^2^) | 74.6 ± 28.6 | 77.6 ± 22.8 | 0.287 |
| Albumin (mg/dL) | 4.6 ± 3.1 | 4.5 ± 0.3 | 0.874 |
| HbA1c (%) | 7.7 ± 1.5 | 8.5 ± 11.5 | 0.448 |
| Fasting blood glucose (mg/dL) | 146.5 ± 56.4 | 135.8 ± 44.0 | 0.051 |
| Postprandial blood glucose (mg/dL) | 233.0 ± 86.6 | 211.8 ± 78.5 | 0.060 |
| Total cholesterol (mg/dL) | 191.0 ± 61.7 | 184.1 ±49.4 | 0.410 |
| Triglyceride (mg/dL) | 464.7 ± 871.0 | 367.3 ± 245.9 | 0.371* |
| HDL cholesterol (mg/dL) | 39.2 ± 8.6 | 42.7 ± 29.8 | 0.390* |
| Uric acid (mg/dL) | 6.2 ± 1.8 | 5.9 ± 1.7 | 0.119 |
| hsCRP (mg/dL) | 7.9 ± 18.3 | 3.8 ± 7.7 | 0.076* |
