# Supplementary material for: Effects of Omega-3 Fatty Acid Supplementation on Diabetic Nephropathy Progression in Patients with Diabetes and Hypertriglyceridemia
Source: PLoS One. 2016 May 2;11(5):e0154683. doi: 10.1371/journal.pone.0154683 (PMC4852914; doi:10.1371/journal.pone.0154683)
Supplement: S2 Table — Data are presented as N(%) or mean ± SD. BMI, body mass index; HDL cholesterol, high density lipoprotein cholesterol; GFR, glomerular filtration rate; ACR, albumin to creatinine ratio; ACE inhibitor, angiotensin-converting enzyme inhibitor; ARB, angiotensin II receptor blocker.*Log transformed. (DOCX) [file pone.0154683.s003.docx]

**S2 table. Baseline characteristics of study subjects according to O3FAs daily dose**

|  | Low dose (<4 g/day) | High dose (≥4 g/day) | P |
| --- | --- | --- | --- |
| Male gender, N (%) | 223 (71.0) | 21 (70.0) | 0.907 |
| Age (year) | 57.1 ± 11.5 | 54.7 ± 14.7 | 0.295 |
| Diabetes duration (year) | 6.7 ± 7.7 | 5.1 ± 7.7 | 0.296 |
| BMI (kg/m^2^) | 25.9 ± 3.3 | 27.6 ± 4.5 | 0.055 |
| Smoking, N (%) | 142 (45.2) | 10 (33.3) | 0.210 |
| Drinking alcohol, N (%) | 161 (51.3) | 12 (40.0) | 0.238 |
| Hypertension, N (%) | 234 (74.5) | 22 (73.3) | 0.887 |
| Fasting blood glucose (mg/dL) | 141.3 ± 50.9 | 140.3 ± 51.0 | 0.918 |
| Postprandial blood glucose (mg/dL) | 221.5 ± 80.1 | 241.7 ± 109.9 | 0.271 |
| Total cholesterol (m/dL) | 185.4 ± 54.2 | 210.0 ± 69.0 | 0.030* |
| Triglyceride (mg/dL) | 401.7 ± 654.4 | 567.6 ± 484.5 | 0.012* |
| HDL cholesterol (mg/dL) | 41.2 ± 22.8 | 38.3 ± 9.8 | 0.374* |
| GFR (mL/min/1.73 m^2^) | 76.4 ± 25.9 | 73.4 ± 25.7 | 0.699* |
| Urine ACR (mg/g) | 428.2 ± 1120.6 | 767.6 ± 2098.6 | 0.329* |
| ACEi/ARB use, N (%) | 226 (72.0) | 21 (70.0) | 0.818 |
| Statin use, N (%) | 198 (63.1) | 13 (43.3) | 0.034 |
| Fenofibrate use, N (%) | 41 (13.1%) | 13 (43.3) | <0.001 |
