# Supplementary material for: Effects of Omega-3 Fatty Acid Supplementation on Diabetic Nephropathy Progression in Patients with Diabetes and Hypertriglyceridemia
Source: PLoS One. 2016 May 2;11(5):e0154683. doi: 10.1371/journal.pone.0154683 (PMC4852914; doi:10.1371/journal.pone.0154683)
Supplement: S3 Table — Reference group = patients with O3FA 1g/day, Model 1: adjusted for age and sex, Model 2: adjusted for age, sex, diabetes duration, body mass index, systolic blood pressure, angiotensin-converting enzyme inhibitor/angiotensin II receptor, and statin use, Model 3: adjusted for age, sex, diabetes duration, body mass index, systolic blood pressure, angiotensin-converting enzyme inhibitor/angiotensin II receptor, statin, fenofibrate, fasting blood glucose, baseline GFR, triglycerides, and total cholesterol. GFR, glomerular filtration rate; O3FAs, omega-3 fatty acids; OR, odds ratio; 95% CI, 95% confidence interval. (DOCX) [file pone.0154683.s004.docx]

**S3**. **Multiple logistic regression analysis to determine variables associated GFR decline in subjects without fenofibrate use**

|  | O3FA 2 g/day |  | O3FA 4 g/day |  |
| --- | --- | --- | --- | --- |
|  | OR (95% CI) | P | OR (95% CI) | P |
| Model 1 | 0.53 (0.32-0.88) | 0.014 | 0.21 (0.06-0.71) | 0.012 |
| Model 2 | 0.51 (0.30-0.87) | 0.013 | 0.16 (0.05-0.57) | 0.005 |
| Model 3 | 0.49 (0.28-0.85) | 0.012 | 0.14 (0.04-0.54) | 0.004 |
